# The Pharmaceutical Industry in 2021. An Analysis of FDA Drug Approvals from the Perspective of Molecules

**DOI:** 10.3390/molecules27031075

**Published:** 2022-02-05

**Authors:** Beatriz G. de la Torre, Fernando Albericio

**Affiliations:** 1 KRISP, College of Health Sciences, University of KwaZulu-Natal, Durban 4001, South Africa; 2 School of Chemistry and Physics, University of KwaZulu-Natal, Durban 4001, South Africa; 3 Institute for Advanced Chemistry of Catalonia (IQAC-CSIC), 08034 Barcelona, Spain; 4 CIBER-BBN, Networking Centre on Bioengineering, Biomaterials and Nanomedicine, Department of Organic Chemistry, University of Barcelona, 08028 Barcelona, Spain

**Keywords:** antibodies, antibody-drug conjugate, API, biologics, CBER, CDER, chemical entities, COVID-19, drug discovery, fluorine-based drugs, natural products, nitrogen aromatic heterocycles, oligonucleotides, pegylation, peptides, TIDES, small molecules

## Abstract

Similar to last year, 2021 will be remembered for the COVID-19 pandemic. Although five vaccines have been approved by the two most important drug regulatory agencies, namely the US Food and Drug Administration (FDA) and the European Medicines Agency (EMA), the pandemic has still not been brought under control. However, despite the context of a global pandemic, 2021 has been an excellent year with respect to drug approvals by the FDA. In 2021, 50 drugs have been authorized, making it the fourth-best year after 2018 (59 drugs) and 1996 and 2020 (53 each). Regarding biologics, 2021 has been the third-best year to date, with 14 approvals, and it has also witnessed the authorization of 36 small molecules. Of note, nine peptides, eight monoclonal antibodies, two antibody-drug conjugates, and two oligonucleotides have been approved this year. From them, five of the molecules are pegylated and three of them highly pegylated. The presence of nitrogen aromatic heterocycles and/or fluorine atoms are once again predominant among the so-called small molecules. This report analyzes the 50 new drugs approved in 2021 from a chemical perspective, as it did for those authorized in the previous five years. On the basis of chemical structure alone, the drugs that received approval in 2021 are classified as the following: biologics (antibodies, antibody-drug conjugates, enzymes, and pegylated proteins); TIDES (peptide and oligonucleotides); combined drugs; natural products; nitrogen aromatic heterocycles; fluorine-containing molecules; and other small molecules.

## 1. Analysis

Last year, we started this analysis by saying that 2020 had been the most difficult year in living memory due to COVID-19 [1], but we expressed hope that the first two vaccines (Pfizer-BioNTech COVID-19 Vaccine and Moderna COVID-19 Vaccine), issued emergency use authorization (EUA) by the Food and Drug Administration (FDA) [2], would allow people to return to some kind of “normality”. However, 2021 (also referred to as “this year” herein) has also been strongly marked by the COVID-19 pandemic. Although an additional vaccine (Janssen COVID-19 Vaccine) was granted EUA by the FDA at the beginning of the year [2], two more (AstraZeneca COVID-19 Vaccine [3] and Novavax COVID-19 vaccine [4]) have been authorized by the European Medicines Agency (EMA), and many countries boast mass vaccination, we are ending the year in some disbelief with respect to a new wave of contagion, being caused by the omicron variant. This situation has kept the pharmaceutical industry alert and prepared over the last few months.

**Figure 1:**
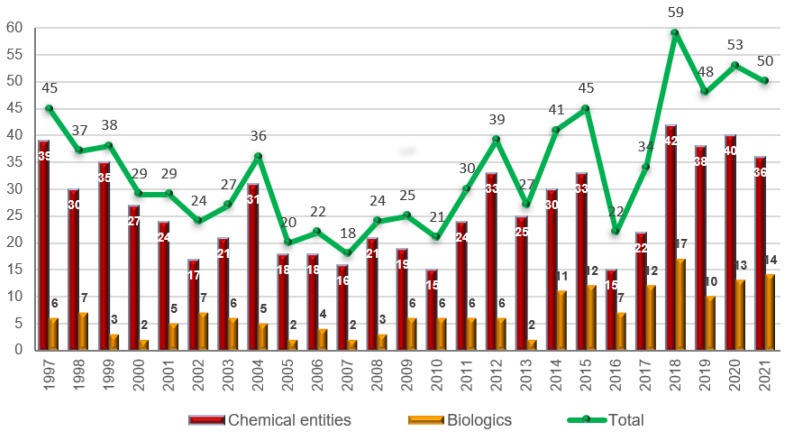
Drugs (New Chemical Entities and Biologics) approved by the FDA in the last 25 years [1,5,6,7,8].

As is easy to imagine, the extraordinary response of the pharmaceutical industry to the pandemic treat cannot overshadow the excellent year that 2021 has been with respect to the approval of new drugs. In this year, the FDA’s Center for Drug Evaluation and Research (CDER) has approved 50 new drugs [5], maintaining the peak started in 2018 with 59 approvals [6], and followed in 2019 and 2020 with 48 and 53 new drugs, respectively [7,8] (210 new drugs in the last four years) (Figure 1). This year’s numbers confirm our earlier expectations and those of other analysts regarding the positive tendency in the number of authorized drugs [1,9,10].

The 50 drugs approved this year comprise 36 New Chemical Entities (NCEs) (40, 38, and 42 in 2020, 2019, and 2018, respectively) and 14 biologics (13, 10, and 17 in 2020, 2019, and 2018, respectively). The figure for 2021 is in full agreement with the number of drugs approved during the last three years (Figure 1). Biologics continue to account for slightly more than 25% of all drugs accepted by the FDA. The year of 2014 marked a change for biologics, as this class of drugs reached double digits; since then, 96 biologics have been approved out of a total of 352 drugs, thus accounting for 27%.

Furthermore, the Center for Biologics Evaluation and Research (CBER) has added 13 new Biological License Application Approvals [11], with full approval of COMIRNATY^TM^, the Pfizer-BioNTech COVID-19 Vaccine being the highlight of 2021.

## 2. Discussion

Fourteen biologics were approved in 2020 (Table 1), of which eight were monoclonal antibodies (mAbs), two Antibody Drug Conjugate (ADCs), two enzymes, and two pegylated biomolecules, namely a hormone and a protein (Table 1).

Although the number of mAbs approved this year was 20% lower than the figure in 2020 (8 vs. 10), mAbs continue to be the most prevalent drug over all types (approximately 15% of the total number of drugs (8 vs. 50)). This year, it is important to highlight an mAb, namely Aducanumab-avwa (Aduhelm^TM^), for the treatment of Alzheimer’s disease; it is considered to be the first drug to directly target the plaques formed by the β-amyloid protein. The approval of this drug has been accompanied by considerable controversy regarding its effectiveness. Although independent studies questioned its efficacy, the drug was approved by the FDA following an accelerated protocol, but the pharmaceutical company was required to undertake a deep clinical phase IV to demonstrate its true effectiveness.

Dostarlimab-gxly (Jemperli^TM^) and amivantamab-vmjw (Rybrevant^TM^) are recommended for cancer; anifrolumab-fnia (Saphnelo^TM^) for lupus, an autoimmune disease; tezepelumab (Tezspire^TM^) for asthma; efgartigimod alfa-fcab (Vyvgart^TM^) for myasthenia gravis (an autoimmune neuromuscular disease); evinacumab-dgnb (Evkeeza^TM^) for familial hypercholesterolemia; and tralokinumab-ldrm (Adbry^TM^) for atopic dermatitis. The mAb approvals in 2021 complement previous authorization of other mAbs for the Ebola virus, eye diseases, the prevention of migraine, psoriasis, HIV, uremic syndrome, and cancer, among others, and validate these molecules as all-round drugs.

In terms of ADCs, 2021 has followed the trends set in previous years with two more approvals, namely tisotumab vedotin-tftv (Tivdak^TM^) and loncastuximab tesirine-lpyl (Zynlonta^TM^), both for cancer, thereby confirming the increasing relevance of this class of drugs. In the last five years (2017–2021), 10 ADCs out of a total of 13 have been approved.

**Figure 2:**
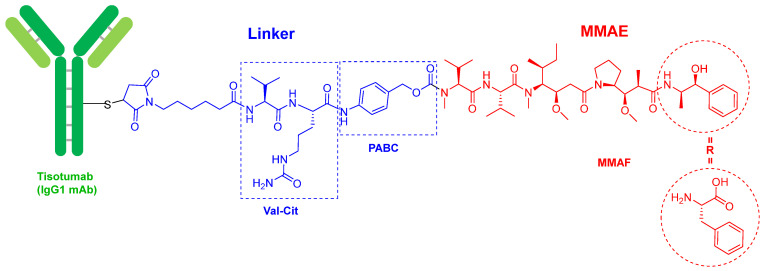
Structure of tisotumab vedotin-tftv.

Tisotumab vedotin-tftv (Figure 2) contains as payload drug monomethyl auristatin E (MMAE), which is an analog of the peptide dolastatin 10, isolated from the Indian Ocean mollusk *Dolabella auricularia*. MMAE is also presented in the following ADCs previously approved by the FDA: enfortumab vedotin-ejfv (Padcev^TM^) and polatuzumab vedotin (Polivy^TM^), approved in 2019, and brentuximab vedotin (Adcetris^TM^, approved in 2015). In fact, the four drugs share the same chemical structure. The mAb is linked through a Cys residue to a maleimide-based linker via a Michael addition. This linker is in turn bound to MMAE via a cleavable dipeptide Val-Cit linker, which contains the self-immolating moiety p-aminobenzyl alcohol carbamate (PABC) at the C-terminus. Belantamab mafodotin-blm (Blenrep^TM^), which was approved in 2020, is very similar to the previous four, but contains monomethyl auristatin F (MMAF) as the cytotoxic. MMAE has a C-terminal moiety, (1S,2R)-2-amino-1-phenylpropan-1-ol, while MMAF has a residue of L-Phe. The natural decapeptide dolastatin 10 has a residue of (S)-dolaphenine (Doe) as C-terminal and an extra N-methyl in the N-terminal derivative of the Val residue, which in the ADCs is linked to PABC. To date, the derivatives of dolastatin 10 are the payloads most used in ADCs (5 over 13).

**Figure 3:**
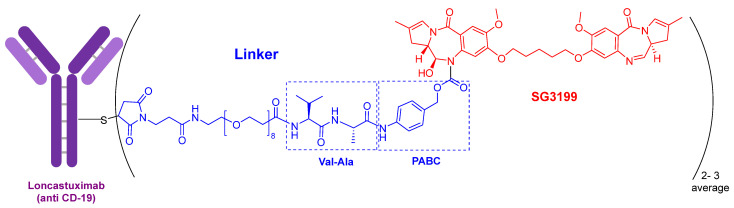
Structure of loncastuximab tesirine-lpyl.

The warhead of loncastuximab tesirine-lpyl (Figure 3) is SG3199, a cytotoxic DNA minor groove interstrand crosslinking pyrrolobenzodiazepine (PBD) dimer. This PBD moiety is also present in anthramycin, which shows by itself very high cardiotoxicity. In this drug, the mAb is also linked through a Cys to maleimide-based moiety, which in turn is linked through a polyethylenglycol (eight ethlylenglycol units) amino acid to the dipeptide Val-Ala and to the PABC. Val-Ala as Val-Cit linker is cleaved by cathepsin once the ADC has entered the tumor cell. The former is more easily produced and shows less aggregation.

Avalglucosidase alfa-ngpt (Nexviazyme^TM^) and asparaginase erwinia chrysanthemi (recombinant)-rywn (Rylaze^TM^) are enzymes. Acid alfpa-glucosidase breaks down glycogen in the lysosome and, in the case of its malfunction, the extra glycogen stored renders Pompe disease, which is characterized by muscle weakness, floppiness, head lag, and even lung infections. Avalglucosidase alfa-ngpt is a recombinant replacement for the endogenous enzyme. Asparaginase erwinia chrysanthemi (recombinant)-rywn) has been approved as part of the treatment for acute lymphoblastic leukemia for patients who have developed hypersensitivity to *E. coli*-derived asparaginase, which was approved in 1978.

Lonapegsomatropin-tcgd (Skytrofa^TM^) and ropeginterferon alfa-2b-njft (Besremi^TM^) are both pegylated biomolecules. The former, a prodrug of somatropin, is a once-a-week treatment for growth hormone deficiency. Ropeginterferon alfa-2b-njft (Aduhelm^TM^) is a new mono pegylated version of interferon alfa-2b, which has also been used for many years to treat polycythemia vera. Interferon alfa-2b requires frequent administration and shows a lack of tolerability. In contrast, ropeginterferon alfa-2b-njft shows a longer half-life and is much better tolerated.

Interestingly, a total of five drugs approved this year are pegylated: three biologics namely those two drugs and loncastuximab tesirine-lpyl, the latter with a defined mini-PEG of eight units; the oligo Casimersen (Amondys 45^TM^) containing a defined mini-PEG of three units; and the peptide pegcetacoplan (Empaveli^TM^). After the withdrawal of peginesatide (Omontys^TM^), a hyperpegylated disulfide cyclic peptide dimer, from the market in 2013, this year’s approvals could mark the renaissance of pegylated drugs.

Although oligonucleotides and peptides (TIDES) are considered chemical entities, they occupy a delimited chemical space between biologics and small molecules. In addition to the MMAE present in an ADC and the two dipeptides (Val-Cit and Val-Ala) present in both ADC linkers, eight peptides and two oligonucleotides have been approved by the FDA in 2021. These figures imply that slightly more than 25% of the new drugs approved (13 vs. 50) are TIDES.

**Figure 4:**
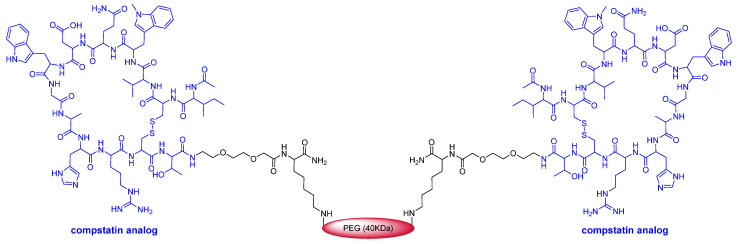
Structure of pegcetacoplan.

This year marks the highest number of peptides ever approved (8). As mentioned above, pegcetacoplan (Empaveli^TM^) (Figure 4) is a highly pegylated peptide with a chemical structure that resembles that of peginesatide. Thus, both molecules contain two copies of a disulfide cyclic peptide (13 aa for pegcetacoplan and 20 aa for peginesatide) and 40 kDa PEG (two units of 20 kDa for peginesatide). Pegcetacoplan is used for the treatment of paroxysmal nocturnal hemoglobinuria, which is characterized by the destruction of red blood cells by complement component 3 (C3), part of the body’s innate immune system. The cyclic peptide is an analog of compstatin (H-Ile-Cys^&^-Val-Val-Gln-Asp-Trp-Gly-His-His-Arg-Cys^&^-Thr-NH_2_) (& indicates the connection between the two Cys), which binds to human C3.

**Figure 5:**
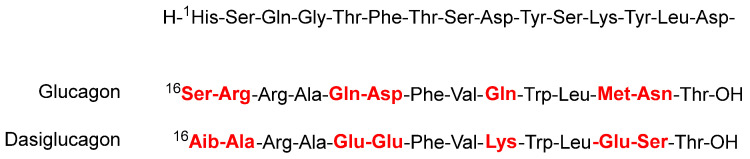
Structure of dasiglucagon.

Dasiglucagon (Zegalogue^TM^) (Figure 5), a ready-to-use 29 aa linear peptide analog of glucagon, is used for the treatment of severe hypoglycemia in diabetes patients. Dasiglucagon is physically and chemically more stable in aqueous media and it shows seven amino acid substitutions compared to endogenous glucagon. All these changes occur in the C-terminal part. Some of the so-called problematic amino acids have been substituted. Thus, the amide residue’s Gln^20^, Gln^24^, and Asn^28^ have been substituted by Glu, Lys, and Ser, respectively, Asp^21^ by Glu, and Met^27^ by Glu. Finally, Ser^16^ and Arg^17^ have been substituted by Aib and Ala, respectively.

**Figure 6:**
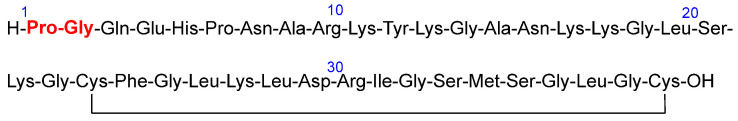
Structure of vosoritide.

Vosoritide (Voxzogo^TM^) (Figure 6), which has been approved for the treatment of achondroplasia (Dwarfism), is an analog of human C-type natriuretic peptide (CNP) that includes 37 aa with two additional resides, Pro and Gly, and the N-terminal. The two Cys residues in the sequence are linked through a disulfide bridge.

**Figure 7:**
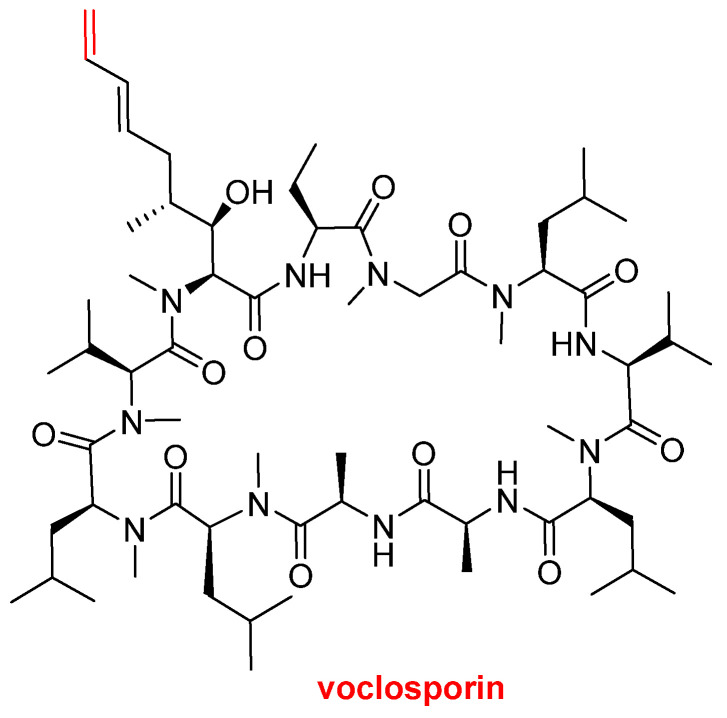
Structure of voclosporin. In red, the difference with cyclosporin is marked: an extra carbon atom linked by a double bond.

Voclosporin (Lupkynis^TM^) (Figure 7), which is an immunosuppressant, has been approved by the FDA for the treatment of lupus nephritis. Voclosporin is an analog of cyclosporine and it has a modification on the β-hydroxy-amino acid. The extended side-chain on this residue with an extra double bond improves potency when compared with the parent cyclosporine.

**Figure 8:**
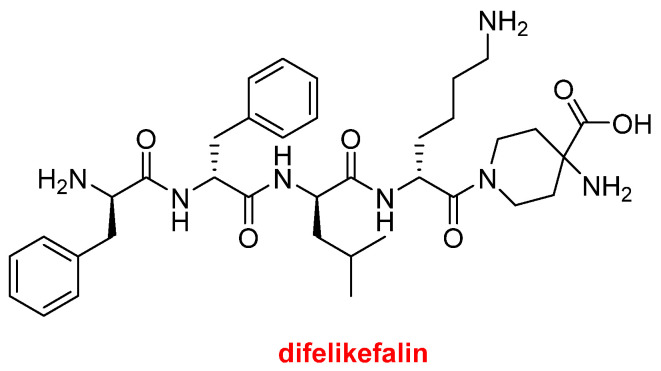
Structure of difelikefalin.

Difelikefalin (Korsuva^TM^) (Figure 8) is an analgesic opioid peptide used to treat pruritus (chronic itchy skin) associated with chronic kidney disease. It is a highly selective agonist of the κ-opioid receptor. Difelikefalin is formed by four D-amino acids (two Phe, one Leu, and one Lys) and the carboxylic group of the D-Lys is bound to 4-aminopiperidine-4-carboxylic acid through its γ amino function.

**Figure 9:**
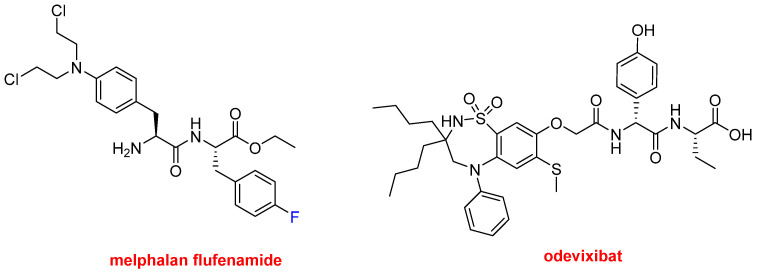
Structures of melphalan flufenamide and odevixibat.

Finally, in this peptide section, three small peptides have also been approved this year. Melphalan flufenamide (Pepaxto^TM^) (Figure 9) is indicated to treat multiple myeloma. It is an ethyl ester dipeptide formed by two Phe derivatives, namely 4-fluoro-phenylalanine and 4-bis(2-chloroethylamine)-phenylalanine. It belongs to a group of alkylating agents developed more than 50 years ago. Specifically, it is an analog of the single amino acid, 4-bis(2-chloroethylamine)-phenylalanine (melphalan). Odevixibat (Bylvay^TM^) (Figure 9) is another dipeptide formed by D-4-hydroxyphenylglycine (N-terminal) and L-ethylglycine (C-terminal), where the N-terminus is acylated with a carboxylic acid moiety containing a dioxidothiadiazepin derivative. Odevixibat was approved for the treatment of pruritus in patients with progressive familial intrahepatic cholestasis.

**Figure 10:**
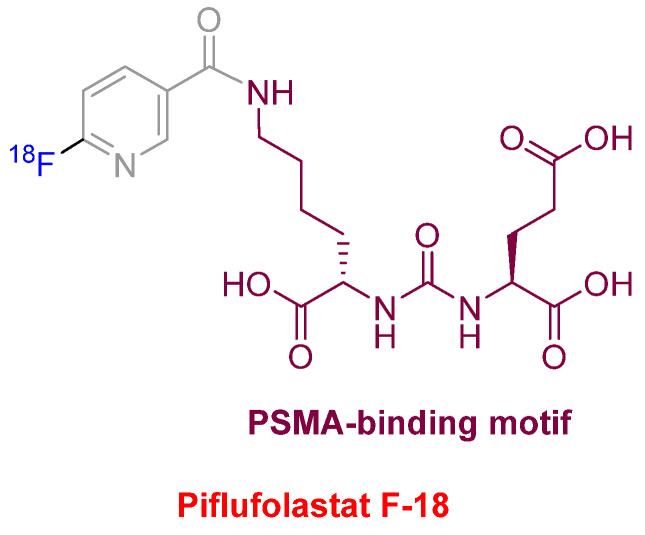
Structure of piflufolastat F-18.

Piflufolastat F-18 (Pylarify^TM^) (Figure 10) was approved for positron emission tomography (PET) imaging as a radioactive diagnostic agent of prostate-specific membrane antigen (PSMA)-positive lesions in men with prostate cancer. This is the second PSMA-targeted PET imaging drug approved after Ga-68 PSMA-1 in the last year. Both drugs share the same PSMA-specific pharmacophore, the urea of the two α-amino functions of L-Glu and L-Lys. In the case of Piflufolastat F-18, the ε-amino function of the Lys is anchored to F-18-labeled 6-fluoronicotinic acid.

**Figure 11:**
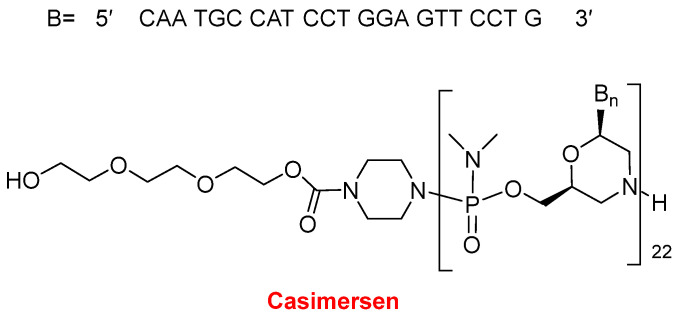
Structure of Casimersen.

In 2021, two oligonucleotide-based drugs were approved by the FDA, following the positive trend initiated in 2016. In the last six years, 11 oligos over a total of 14 have reached the market. Casimersen (Amondys 45^TM^) (Figure 11) is an antisense oligonucleotide of phosphorodiamidate morpholino oligomer (PMO) indicated for Duchenne muscular dystrophy (DMD). This is the fourth PMO approved for the treatment of DMD, after eterlipsen, golodirsen, and viltolarsen in 2016, 2019, and 2020, respectively. While the first two are for patients with genetic mutations subject to exon 53 skipping of the dystrophin gene, and the third one is for patients with mutations skipping exon 51, casimersen is amenable to those with mutations skipping exon 45. The sequence of 22 mer contains 5 units of 5-methyluracil and the 5′ ends with a mini-PEG of 3 units, a moiety unique among the four oligonucleotides marketed for DMD.

**Figure 12:**
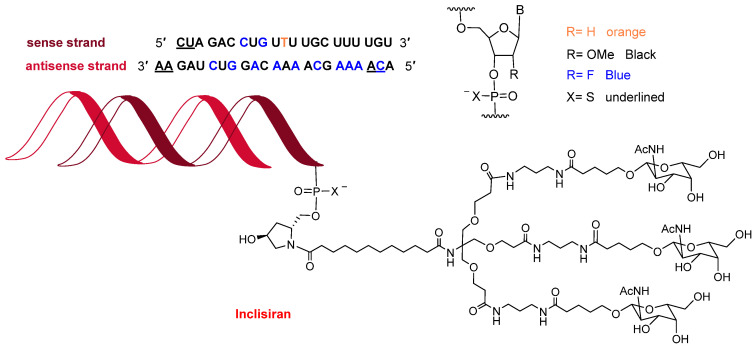
Structure of inclisiran.

Towards the end of the year, the second oligonucleotide, inclisiran (Leqvio^TM^) (Figure 12), was approved for the treatment of clinical atherosclerotic cardiovascular disease or familial hypercholesterolemia. The chemical structure of inclisiran is very similar to that of lumasiran and givosiran, which were approved last year for the treatment of hyperoxaluria type 1 and in 2019 for acute hepatic porphyria, respectively. Inclisiran is a double-stranded siRNA—with 21 and 23 ribonucleosides for the sense and antisense strands, respectively. It has a total of six tiophosphate linkages—the same as lumasiran and givosiran—as well as 12 2′-F-ribonucleoside units (10 and 16 in lumasiran and givosiran, respectively) to improve the stability of the double-strand. The remaining ribonucleosides are 2′-methoxy, except one deoxythymine (dT). Similar to lumasiran and givosiran, inclisiran is presented through an Enhanced Stabilization Chemistry (ESC), which, in addition to F- and methoxy-ribonucleosides, has the 3′ end of the sense strand linked to a short dendrimer bearing N-acetylgalactosamine (GalNAc), which mediates the binding and internalization of the drug by hepatocytes. The six thiophosphates are present in the other three ends—two in each. This kind of structure is also present in lumasiran and givosiran.

**Figure 13:**
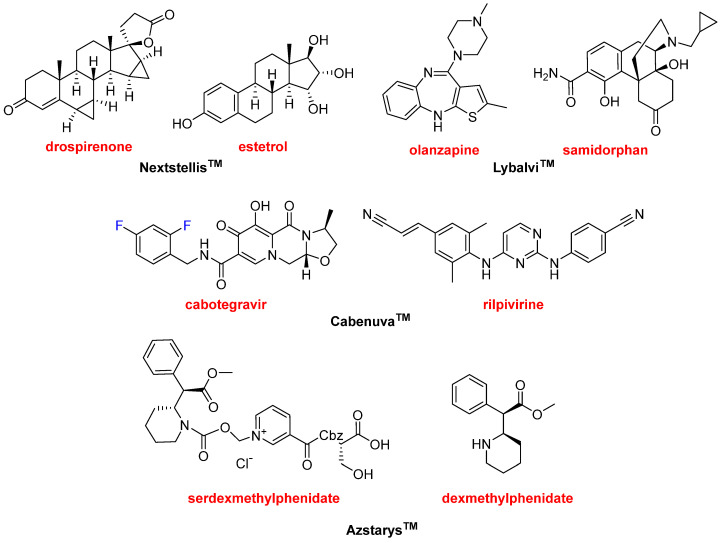
Structure of the combination drugs: Nextstellis^TM^ (drospirenone/estetrol); Lybalvi^TM^ (olanzapine/samidorphan); Cabenuva^TM^ (cabotegravir/rilpivirine); and Azstarys^TM^ (serdexmethylphenidate/dexmethylphenidate).

The year 2021 has witnessed the approval of four drugs that contain more than one Active Pharmaceutical Ingredient (API). This confirms the trend over the last few years, where 12 combination drugs have been approved in the period 2016–2020. Nextstellis^TM^ (Figure 13) is a fixed-dose combination of drospirenone and estetrol used as pregnancy prevention. Drospirenone could be considered an “old” drug (patented in 1976) and it can be administered alone under the brand Slynd^TM^ or in combination with strogens, as it is in this case. Estetrol, which is even older (discovered in 1965) than drospirenone, is a weak estrogen steroid hormone.

Lybalvi^TM^ (Figure 13) is a combination of olanzapine and samidorphan used to treat schizophrenia and bipolar disorder. Olanzapine, which is a benzodiazepine, is also an “old” drug marketed under the name of Zypreza^TM^ with basically the same indications as Lybalvi^TM^. Samidorphan is an opioid antagonist that reduces the weight gain associated with olanzapine. Olanzapine and samidorphan form, separately or in combination, part of other fixed-dose combinations currently being evaluated.

Another combination for the treatment of HIV has been approved this year, supporting the tendency towards the use of retroviral cocktails to treat this condition. The Cabenuva^TM^ package (Figure 13) contains two separate injection vials, one with cabotegravir and the other with rilpivirine. Cabotegravir is already marketed under the name of Vocabria^TM^. Rilpivirine is also sold under the names of Edurant^TM^ and Rekambys^TM^. Rilpivirine is also part of several fixed-dose medications. Thus, the combination of rilpivirine with emtricitabine and tenofovir disoproxil was approved by the FDA in 2011 as Complera^TM^. Similarly, combined rilpivirine with emtricitabine and tenofovir alafenamide was approved by the FDA as Odefsey^TM^ in 2016. Combined administration of rilpivirine and dolutegravir (Juluca^TM^) was approved in 2017 by the FDA. Azstarys^TM^ (Figure 13) is a combination of serdexmethylphenidate and dexmethylphenidate for the treatment of attention deficit hyperactivity disorder. Dexmethylphenidate is already sold for the same indication as Focalin^TM^ and it is one of the drugs most commonly prescribed in the USA.

**Figure 14:**
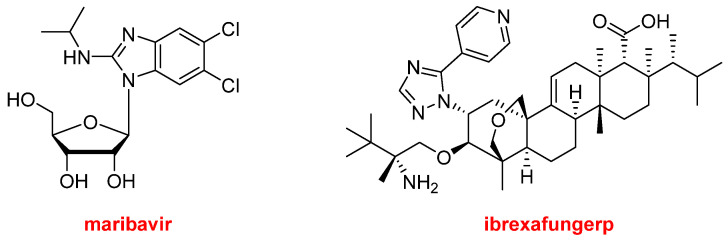
Structures of maribavir and ibrexafungerp, both drugs inspired by natural products.

Regarding natural product-based drugs or those directly inspired by natural products, the 2021 harvest has not been abundant. Thus, in addition to the two steroids, drospirenone and estetrol, present in Nextstellis^TM^, and the morphine derivative, samidorphan, which is part of Lybalvi^TM^, only two more drugs could be considered part of the natural product class. Maribavir (Livtencity^TM^) (Figure 14), which is a ribose, is an antiviral drug used to treat post-transplant cytomegalovirus. Ibrexafungerp (Brexafemme^TM^) (Figure 14) is a triterpenoid antifungal drug indicated for vulvovaginal candidiasis (vaginal yeast infection).

Similar to previous years, 2021 has witnessed the authorization of a large number of drugs that contain fluorine atoms and/or nitrogen aromatic heterocycles. Both chemical moieties are common among the drugs approved by the FDA every year. In addition to piflufolastat F-18 and inclisiran, seven additional drugs contain F. In fact, all seven drugs contain more than one of this atom.

**Figure 15:**
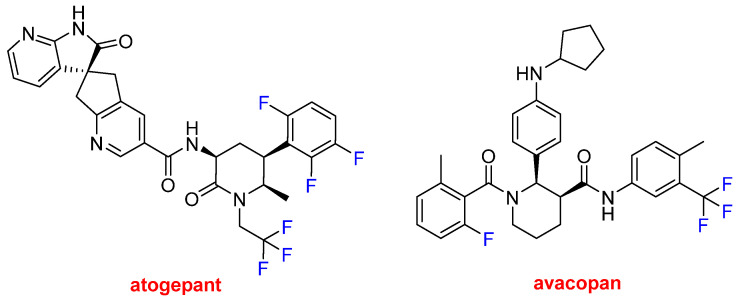
Structures of atogepant and avacopan, both of which contain trifluoromethyl moieties.

Atogepant (Qulipta^TM^) and avacopan (Tavneos ^TM^) (Figure 15) each contain a trifluoromethyl moiety and F atoms bound to aromatic rings. Atogepan is used to treat migraines, while avacopan was approved for anti-neutrophil cytoplasmic autoantibody-associated vasculitis, which is a rare autoimmune disease. Some analysts anticipate that avacopan and future drugs could replace toxic steroid-based treatments.

**Figure 16:**
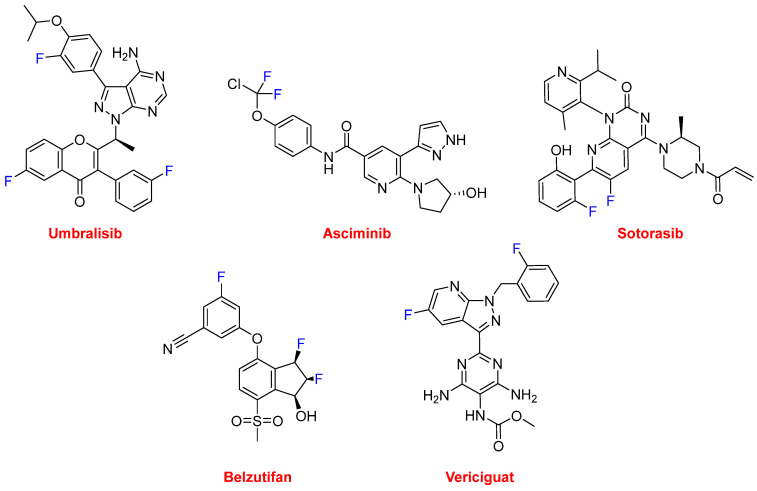
Structures of umbralisib, asciminib, sotorasib, belzutifan, and vericiguat.

Several of these F-containing drugs have been authorized for the treatment of various types of cancer. Thus, umbralisib (Ukoniq^TM^) (Figure 16) is recommended for the treatment of follicular lymphoma and marginal zone lymphoma. Umbralisib is a kinase inhibitor, including PI3K-delta and casein kinase CK1-epsilon. Asciminib (Scemblix^TM^) (Figure 16), which is also a kinase inhibitor, has been approved for the treatment of Philadelphia chromosome-positive chronic myeloid leukemia. From a structural point of view, umbralisib contains a trihalogeno moiety with two F atoms and one Cl atom. Sotorasib (Lumakras^TM^) (Figure 16) is indicated to treat non-small-cell lung cancer (NSCLC), and it targets a specific mutation in the KRAS protein, which is associated with the development of various forms of cancer. Belzutifan (Welireg^TM^) (Figure 16) has been approved for the treatment of von Hippel-Lindau disease-associated renal cell carcinoma. It is considered first in class and has been the first inhibitor of hypoxia-inducible factor-2 alfa to be authorized. Vericiguat (Verquvo^TM^) (Figure 16) is indicated for the mitigation of the risk of cardiovascular death and hospitalization for chronic heart failure.

From a structural point of view, drugs containing nitrogen aromatic heterocycles are the most common on the market. In 2021, in addition to the peptide piflufolastat F-18; the two oligonucleotides, casimersen and inclisiran; cabotegravir and rilpivirine, both in Cabenuva^TM^; serdexmethylphenidate in Azstarys^TM^; olanzapine in Lybalvi^TM^; the natural products-inspired maribavir and ibrexafungerp; and five of the F classified drugs (atogepan, umbralisib, asciminib, sotorasib, and vericiguat), ten more drugs containing nitrogen aromatic heterocycles have been approved. Therefore, almost 50% of all drugs (23 vs. 50) are nitrogen aromatic heterocycles.

**Figure 17:**
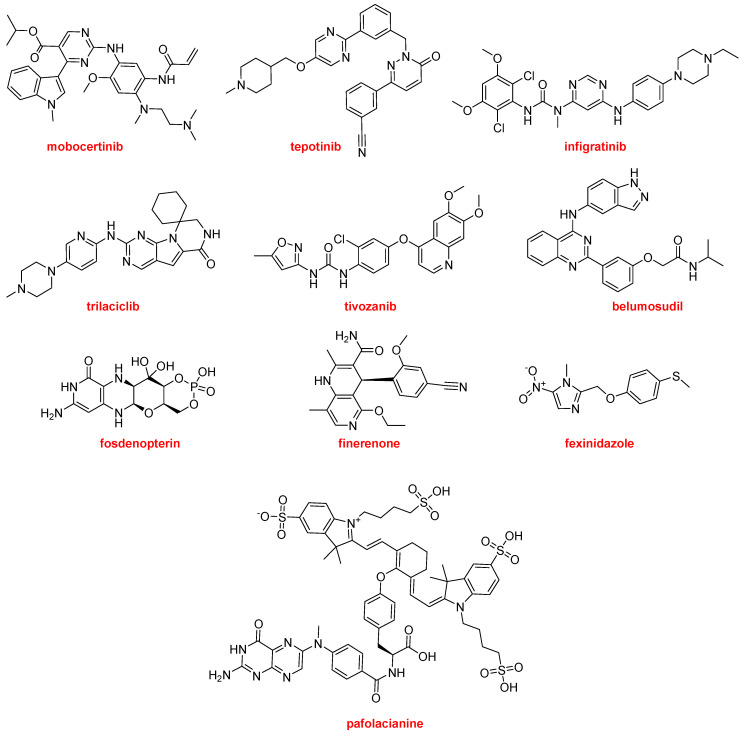
Structures of mobocertinib, tepotinib, infigratinib, trilaciclib, tivozanib, belumosudil, fosdenopterin, finerenone, fexinidazole, and pafolacianine, all of which contain nitrogen aromatic heterocycles.

The most common moiety among the nitrogen aromatic heterocycles presented in the drugs approved this year is pyrimidine and more concretely aminopyrimidine. Mobocertinib (Exkivity^TM^) is an irreversible tyrosine kinase inhibitor that forms a covalent bond with a Cys residue present in the epidermal growth factor receptor (EGFR). The position of this residue is rare in other kinases. Mobocertinib is recommended for the treatment of NSCLC. Tepotinib (Tepmetko^TM^) is also a tyrosine kinase inhibitor and is indicated for NSCLC tumors with a particular mutation. Another kinase inhibitor for the treatment of cholangiocarcinoma (bile duct cancer) is infigratinib (Truseltiq^TM^). Trilaciclib (Cosela^TM^), another kinase inhibitor, is a medication used to mitigate chemotherapy-induced myelosuppression in small cell lung cancer. Tivozanib (Fotivda^TM^), a VEGF receptor tyrosine kinase inhibitor, has been authorized for the treatment of refractory or relapsed advanced renal cell carcinoma. Belumosudil (Rezurock^TM^) is a serine/threonine kinase inhibitor indicated for the treatment of chronic graft versus host disease, a condition that might occur after a bone marrow transplant to fight cancer. Fosdenopterin (Nulibry^TM^) is the first drug approved by the FDA for the treatment of molybdenum cofactor deficiency type A, a rare genetic disease. Finerenone (Kerendia^TM^) is indicated for reducing the risk of kidney and heart complications in chronic kidney disease associated with type 2 diabetes. The nitroimidazole fexinidazole (Fexinidazole^TM^) is an “old drug” that was described in 1978 and approved by the FDA this year to treat the sleeping sickness (African trypanosomiasis) caused by the parasite Trypanosoma brucei gambiense. Pafolacianine (Cytalux^TM^) is the second optical imaging agent, after piflufolastat F-18, to be approved this year. Pafolacianine is employed for the identification of ovarian cancer lesions (Figure 17).

**Figure 18:**
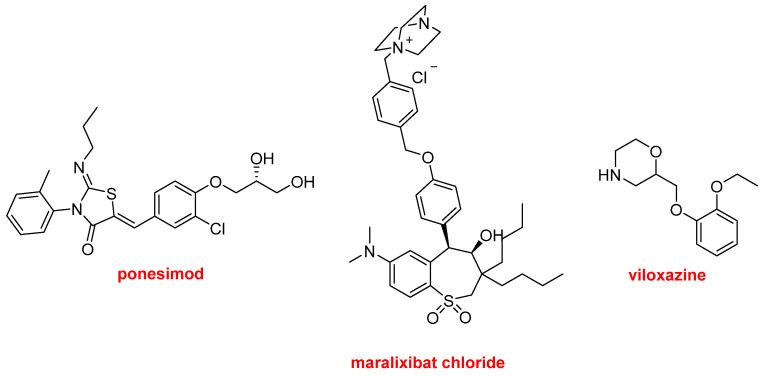
Structure of ponesimod, maralixibat chloride, and viloxazine.

Finally, Ponesimod (Ponvory^TM^) has been approved for the treatment of multiple sclerosis. Maralixibat chloride (Livmarli^TM^) has been authorized for the treatment of cholestatic pruritus associated with Alagille syndrome. Viloxazine (Qelbree^TM^) is a racemic compound that has been available in the European market as an antidepressant for more than two decades and has been repurposed as a treatment for attention deficit hyperactivity disorder this year (Figure 18).

## 3. Conclusions and Perspectives

The outbreak of COVID-19 in 2020 has posed and continues to pose a serious threat to public health. However, a year ago, there was a shared hope that the two vaccines granted EUA by the FDA and those to be authorized in the following months would allow mass vaccination and with it the end of the pandemic. The vaccination process has not been homogeneous within the same country or globally. In a somewhat irrational way, strong anti-vax movements have emerged worldwide. COVID-19 is a global problem, and therefore, it deserves global responses. Unequal vaccination both globally and regionally is favoring the appearance of new variants of the virus, such as omicron, and with them the persistence of the pandemic.

In the context of COVID-19, during 2021, the strength of the pharmaceutical ecosystem, which in addition to big pharma and biotech companies also includes hospital and academic groups, contract research, manufacturing organizations, and regulatory agencies, has been reflected by the drugs granted EUA by the FDA [12]. At the end of this year (December 2021), the retrovirals molnupiravir (a nucleoside) and paxlovid (a co-package containing nirmatrelvir and ritonavir) received authorization for the treatment of mild to moderated COVID-19 with high risk to progress to severe status, which can end in hospitalization and/or death. In addition, several mAbs, such as tocilizumab, sotrovimab, and the co-administration of bamlanivimab and etesevimab, have received EUA for COVID-19 treatment (mild to moderated), and evusheld (co-package of tixagevimab with cilgavimab) has been authorized as a prophylactic for the prevention of the infection. In this regard, 2022 should witness final approval of some of these drugs by the FDA.

Despite the turmoil that COVID-19 has caused in the pharmaceutical sector, 2021 has once again been an excellent year in terms of new drugs accepted by the FDA, similar to 2020 and the preceding years. Since 2018, there has been an average of approximately 50 drugs approved every year, thereby confirming the predictions of analysts in recent years [1,9,10]

**Figure 19:**
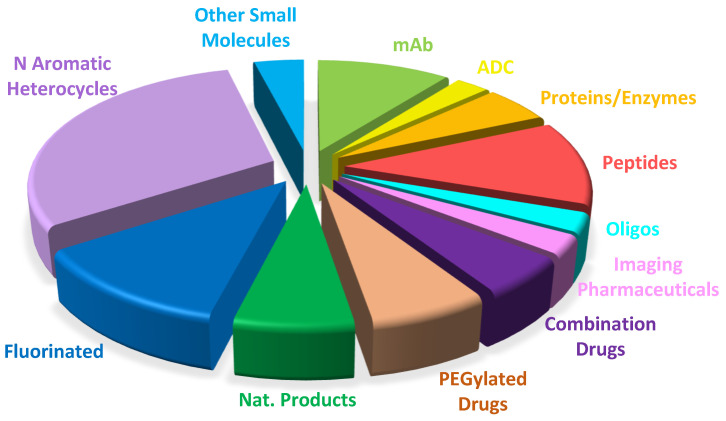
Drugs approved by the FDA in 2021 classified on the basis of chemical structure (drugs can belong to more than a class).

Figure 19 shows the FDA drug approvals in 2021 classified on the basis of chemical structure.

Again, in 2021, biologics have maintained double-digit status and the 14 approved drugs this year make it the second-best harvest of this drug class after 2018, when 17 were authorized. Eight mAbs were approved following the ten, five, eleven, and nine approved in 2020, 2019, 2018, and 2017, respectively. Again, similar to 2020, two ADCs were approved this year. One of them, tisotumab vedotin-tftv, contains a synthetic analog of the sea peptide dolastatin 10 as warhead. With this drug, five out of thirteen ADCs to have received approval by the FDA contain analogs of this peptide. The second ADC approved this year contains three differentiated moieties, a pyrrolobenzodiazepine-type dimer as cytotoxic payload, the dipeptide Val-Ala as part of the cleavable linker, and an eight etlylenglycol unit to confer solubility. Interestingly, two more biologics, lonapegsomatropin-tcgd and ropeginterferon alfa-2b-njft, and one peptide, pegcetacoplan, are highly pegylated, and the oligo casimersen has a three etlylenglycol unit. Could these drugs signify the return of pegylated drugs after the withdrawal of peginesatide from the market in 2013?

In addition to the analog of dolastatin 10 present in the ADC Tisotumab vedotin-tftv, and the two dipeptides Val-Ala and Val-Cit present in both ADCs, eight peptide drugs have been approved in 2021. This means that approximately 20% of drugs approved this year are peptides. From a structural point of view, the 2021 peptide-based drug harvest shows great diversity. In addition to pegcetacoplan, which is pegylated and contains two copies of a 13 aa disulfide bridge peptide, the harvest contains large peptides, such as vosoritide (37 aa), which also contains a disulfide bridge, dasiglucagon (29 aa), and homodetic cyclic peptides such as voclosporin, and then small peptides of five and two amino acids.

Regarding oligos, 2021 has been a xerox copy of 2020 [1]. In both years, two oligos were approved, one antisense PMO and one double-stranded siRNA. With the approvals of this year, four drugs of each class have now been authorized by the FDA. Given this constant trickle of oligos onto the market and the existing pipeline, many more drugs belonging to this class are expected to become available in the next few years.

Although there is no doubt that natural products are an important source of inspiration in the drug discovery field, 2021 cannot be considered a good year for them, as only four drugs contain or were inspired by natural products.

The presence of nitrogen aromatic heterocycle- and fluorine-based drugs continue to be a constant. Thus, if biologics and peptides are excluded from the analysis, 82% of the drugs approved this year contain the first moiety and 33% the second.

Oncology continues to be the main target of the drugs approved by the FDA in 2021—with four of them indicated for metastatic NSCLC, kinase inhibitors being the main target. Two imaging agents have been approved this year, an optical one and the second radiolabeled with F18.

We consider that our honorific title “Molecule of the Year 2021” should be shared by an oligo, the double-stranded siRNA inclisiran, used to treat familial hypercholesterolemia and with an important impact on arteriosclerosis, and an mAb, aducanumab-avwa, intended for the treatment of Alzheimer’s disease. Both drugs are called upon to change the map of the two diseases they are indicated for.

## Figures and Tables

**Table 1:** Biologics approved by the FDA in 2021 [5].

Trade Name ^a^	Active Ingredient ^a^	Class	Indication
Adbry^TM^	Tralokinumab-ldrm	Monoclonal antibody	Atopic dermatitis
Aduhelm^TM^	Aducanumab-avwa	Monoclonal antibody	Alzheimer’s disease
Besremi^TM^	Ropeginterferon alfa-2b-njft	Pegylated Protein	Polycythemia vera (overproduction of red blood cells)
Evkeeza^TM^	Evinacumab-dgnb	Monoclonal antibody	Homozygous familial hypercholesterolemia
Jemperli^TM^	Dostarlimab-gxly	Monoclonal antibody	Endometrial cancer
Nexviazyme^TM^	Avalglucosidase alfa-ngpt	Enzyme	Glycogen storage disease type II (Pompe disease)
Rybrevant^TM^	Amivantamab-vmjw	Monoclonal antibody	Non-small cell lung cancer
Rylaze^TM^	Asparaginase erwinia chrysanthemi (recombinant)-rywn	Enzyme	Acute lymphoblastic leukemia and lymphoma
Saphnelo^TM^	Anifrolumab-fnia	Monoclonal antibody	Systemic lupus erythematosus
Skytrofa^TM^	Lonapegsomatropin-tcgd	Pegylated hormone	Growth hormone deficiency
Tezspire ^TM^	Tezepelumab	Monoclonal antibody	Asthma as maintenance therapy
Tivdak^TM^	Tisotumab vedotin-tftv	Antibody-drug conjugate	Cervical cancer
Vyvgart^TM^	Efgartigimod alfa-fcab	Monoclonal antibody (fragment)	Myasthenia gravis
Zynlonta^TM^	Loncastuximab tesirine-lpyl	Antibody-drug conjugate	Relapsed or refractory large B-cell lymphoma

^a^ Trade name used in the USA.

## Data Availability

Not applicable.
